# Using LS-SVM Based Motion Recognition for Smartphone Indoor Wireless Positioning

**DOI:** 10.3390/s120506155

**Published:** 2012-05-10

**Authors:** Ling Pei, Jingbin Liu, Robert Guinness, Yuwei Chen, Heidi Kuusniemi, Ruizhi Chen

**Affiliations:** Department of Navigation and Positioning, Finnish Geodetic Institute, FIN-02431 Masala, Finland; E-Mails: jingbin.liu@fgi.fi (J.L.); robert.guinness@fgi.fi (R.G.); yuwei.chen@fgi.fi (Y.C.); heidi.kuusniemi@fgi.fi (H.K.); ruizhi.chen@fgi.fi (R.C.)

**Keywords:** motion recognition, LS-SVM, indoor navigation, positioning, wireless, smartphone

## Abstract

The paper presents an indoor navigation solution by combining physical motion recognition with wireless positioning. Twenty-seven simple features are extracted from the built-in accelerometers and magnetometers in a smartphone. Eight common motion states used during indoor navigation are detected by a Least Square-Support Vector Machines (LS-SVM) classification algorithm, e.g., static, standing with hand swinging, normal walking while holding the phone in hand, normal walking with hand swinging, fast walking, U-turning, going up stairs, and going down stairs. The results indicate that the motion states are recognized with an accuracy of up to 95.53% for the test cases employed in this study. A motion recognition assisted wireless positioning approach is applied to determine the position of a mobile user. Field tests show a 1.22 m mean error in “Static Tests” and a 3.53 m in “Stop-Go Tests”.

## 1. Introduction

Nowadays, with the explosive growth of the capabilities in handheld devices, various components are embedded into smartphones, such as GPS, WLAN (a.k.a. Wi-Fi), Bluetooth, accelerometers, magnetometers, cameras, *etc.* Because of their locating capabilities, which are quickly becoming one of the standard features in mobile devices, more and more people are getting used to the location-enabled life. Employing Global Navigation Satellites Systems (GNSS), the applications in the “smart” devices can greatly enrich the end users' outdoor activities. However, given the nature of GNSS design, they are clearly not well-suited for applications in urban canyons and indoor environments. Satellite-based positioning technologies continue to struggle indoors, due to well known issues, such as the weak signal or non-line-of-sight (NLOS) conditions between the mobile user and satellites.

To address positioning and navigation in GNSS-degraded or denied areas, various technologies are broadly researched [1]. Most research topics focus on the high-sensitivity GNSS [2], optical navigation systems [3,4], ultrasound solutions [5], WLAN [6], Bluetooth [7,8], Zig Bee [9], Ultra Wide Band [10], cellular networks [2], RFID [11], magnetic localization [12], inertial measurement units [13,14], signals of opportunity [15], biosensor [16,17], and also hybrid solutions [18–21].

Benefiting from the existing infrastructure, RF-based technologies, such as WLAN, Bluetooth, cellular network, and RFID, are definitely one of highest potential alternatives. RADAR [6] was one of the first WLAN-based positioning systems to compute the mobile device's location based on radio signal strength (RSS) from many access points (APs). Skyhook wireless is a system that depends on information about the AP's coordinates in a database in order to predict location [22]. Ekahau [23] provides an easy and cost-effective solution for locating people, assets, inventory and other objects using Wi-Fi. The Active Badge [24] system uses ceiling-mounted infrared sensor detectors to detect signals from a mobile active badge. Place Lab [25] has even more ambitious goals as seeking to create a comprehensive location database that uses fixed-commodity Wi-Fi, GSM and Bluetooth devices as global beacons.

Meanwhile, human physical activity recognition using MEMS sensors has been extensively applied for health monitoring, emergency services, athletic training, navigation, *etc.* [26,27]. Since motion sensors such as accelerometers, gyroscopes and magnetometers are integrated into a smartphone, they bring the opportunity to assist navigation with knowledge about the motion of a pedestrian [28,29].

Since mobile devices are becoming smarter and smarter nowadays, the smartphone already contains the potential for indoor navigation and positioning within the existing infrastructures [19]. This paper presents an indoor pedestrian navigation solution relying on motion recognition in an office environment utilizing the existing WLAN infrastructure.

## 2. Motivation

Related research indicates that utilizing opportunistic signals of, e.g., WLAN, is an efficient locating alternative in GPS-denied environments. However, in order to minimize a smartphone's battery drain, the WLAN scanning interval is always limited. For instance, most of the Nokia mobile phones refresh the scanned WLAN information proximately every 8–10 s. The default scanning interval of most Android devices is 15 s. On the other hand, other built-in sensors such as accelerometers are always turned on, in order that the physical orientation of the smartphone is always known to the system. These sensors provide an alternative for positioning while WLAN positioning is unavailable.

During the gaps where no wireless signal is updated, the most essential elements for navigation are the movement speed and orientation (*i.e.*, heading). As long as they are determined, it is possible to estimate the position of the user every second using dead-reckoning. Therefore, this paper presents a method to use the built-in tri-axial accelerometer and magnetometer on a smartphone to recognize the user's movement parameters. The proposed solution detects the physical movements using simple acceleration and orientation features throughout the navigation process. With the recognized motions, it is possible to reasonably estimate the speed and position over the period between wireless scans.

Human motion has been widely studied for decades, especially in recent years using computer vision technology. Poppe gives an overview of vision based human motion analysis in [30]. Aside from vision-based solutions, sensor-based approaches are also extensively adopted in biomedical systems [31–34]. Most of the previous motion recognition related research assumed that the Micro-Electro-Mechanical Systems (MEMS) inertial sensors used are fixed on a human body [35–38] (e.g., in a pocket, clipped to a belt or on a lanyard) and that an inference model can be trained according to a handful of body positions. Some of them use phones as a sensor to collect activities for off-line analysis purposes [39]. Compared to the daily activities, such as “Sitting”, “Walking”, “Running”, “Jumping”, the motions of a pedestrian who is using a smartphone for navigation in three-dimensional indoor structures are far more complicated due to the arbitrary gestures while a phone is kept in hand. Hence this paper primarily focuses on the possible motion states of a user with a phone in hand while navigating. References [28] and [29] have briefly presented the preliminary results of our previous research in this topic.

## 3. Motion States

Unlike the solution with sensors fixed on the body, a smartphone in hand has more degrees of freedom (DOF) during the navigation process. Even if we only consider the case where the user holds the phone in hand, the motion behaviour is still complicated. For this reason, we defined eight most common motion states during pedestrian navigation in this paper. In order to classify the motion states, twenty-seven features are investigated in this section.

### 3.1. Motion Definition

**Figure 1.**
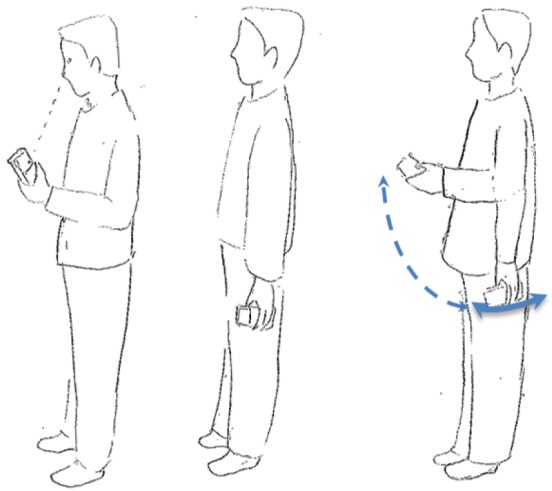
S-series (left and middle: ST, right: SS).

**Figure 2.**
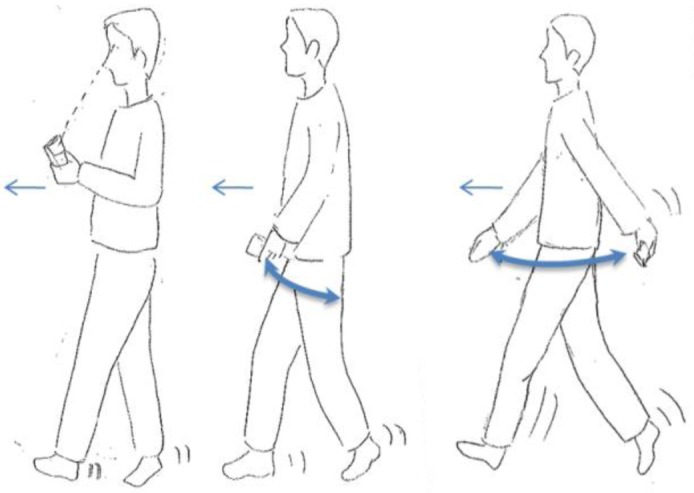
W-series (left: WH, middle: WS, and right: WF).

**Figure 3.**
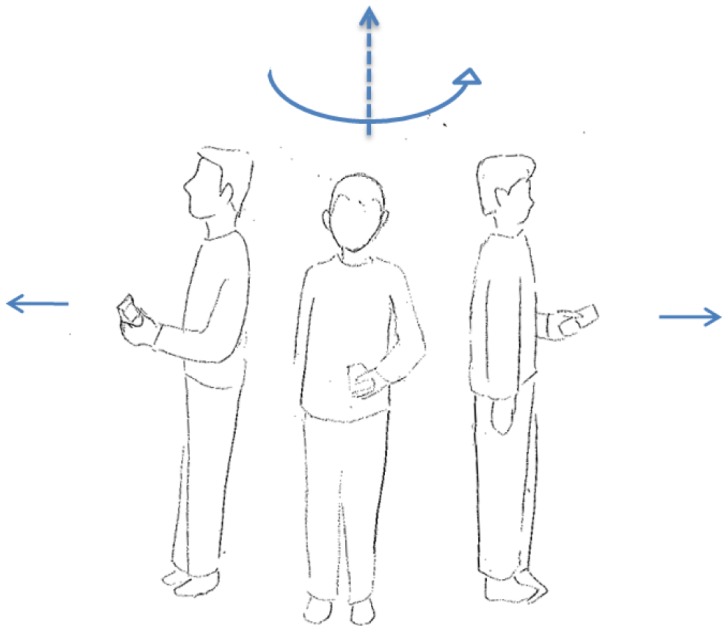
T-series (UT).

**Figure 4.**
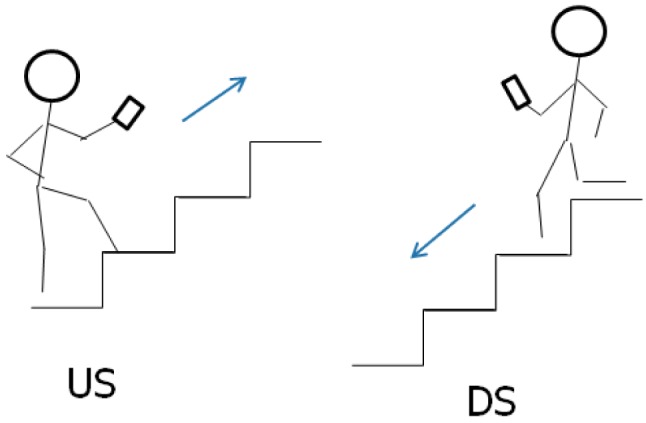
V-series (left: US and right: DS).

The motion states, as defined in Table 1, are grouped into four series as follows:

1. S-series motion states (Figure 1) refer to the stationary behavior during a navigation process. ST is a state where a user keeps a phone in hand without any movement. In contrast, SS is a category of the movement where user's location does not change, but the phone is moving in a swinging.
2. W-series is relevant to walking. After observing the walking behaviour of the user when navigating, three types of walking motion states have been defined. As shown in the left image of Figure 2, WH represents the motion state where the user is using the navigation application on the handset while walking. The user often keeps his or her eyes on the screen of a smartphone in this state. WS stands for the normal walking behaviour, when the user is not using the navigation application but is holding the smartphone in his or her hand. As the center image of Figure 2 indicates, a small arm swinging motion exists when the user is walking in normal speed, while the right image of Figure 2 shows the WF state, which represents a fast walking behaviour with significantly arm swinging.
3. T-series is related to turning motions. UT represents so-called U-turning, which is a spot turn without any horizontal displacement. As shown in Figure 3, a UT motion results in a heading change of 180° after turning.
4. V-series concerns motions in the vertical dimension. In Figure 4, US and DS are going up/down the stairs, respectively.

### 3.2. Feature Definition

**Figure 5.**
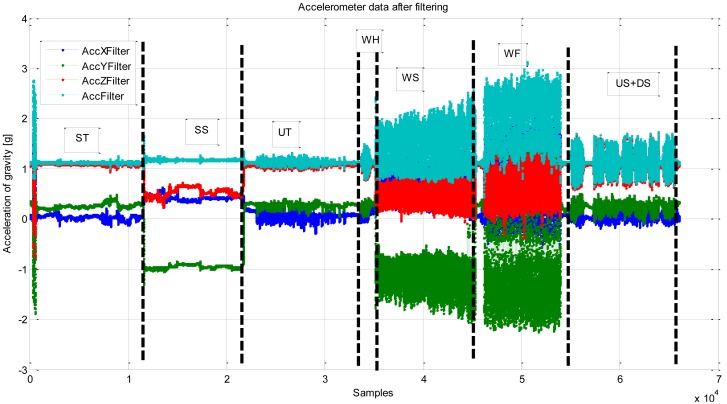
Accelerometer readings.

When using tri-axis accelerometer sensors, the sensor orientation determines the local coordinate system of each (x, y, z) reading. Most previous research work on motion recognition has used body-worn accelerometer sensors, *i.e.*, sensors attached to the body in a constrained orientation. When smartphones are used as portable sensors, the orientation of the sensors is not known *a priori*. Figure 5 gives a typical example of the accelerometer readings after filtering from a smartphone while different motion states are performed.

To avoid this orientation problem, the magnitude of the accelerometer signal (see the ‘Acc Filter’ line of Figure 5) can be used. This solution, however, will cause the three-directional acceleration information to be lost. Therefore, we use an estimated gravity vector as a reference to compute the dynamics of the smartphone's orientation. The gravity vector is trained with the data obtained in the static status (see the left image of Figure 1) in a calibration phase (e.g., 10 s). When the estimated gravity vector is known, it enables the estimation of the vertical component and the magnitude of the horizontal component of a user's motion [39].

The gravity vector is denoted as:

(1) $$G=(g_{x},g_{y},g_{z})$$

where *g_x_, g_y_, g_z_* are the mean values of the gravity vector along the respective axes over the calibration phase.

The acceleration vector can be expressed as:

(2) $$A=(a_{x},a_{y},a_{z})$$

where *a_x_, a_y_, a_z_* are the accelerometer readings along the respective axes at the given time.

The projection of *A* (denoted as *A_p_*) onto the gravity vector *G* can be calculated as the vertical component inside of *A*. The vertical component of *A* can be calculated as the projection of *A* onto the gravity vector *G*:

(3) $$A_{p}=\left(\frac{A•G}{G•G}\right)G$$

Then the horizontal component *A_h_* can be computed using vector subtraction:

(4) $$A_{h}=A-A_{p}$$

The direction of *A_h_* relative to the horizontal axis in the global 3-axis coordinate system, however, still cannot be obtained. For this reason, we use the magnitude of the acceleration in the horizontal plane, which is orthogonal to the estimated gravity vector *G*. In this paper, the mean of the acceleration in the horizontal plane (MeanAccH), variance of the acceleration in the horizontal plane (VarAccH), and the variance of the magnitude of the acceleration (VarAcc) are selected as the simple features to detect the motion states.

**Figure 6.**
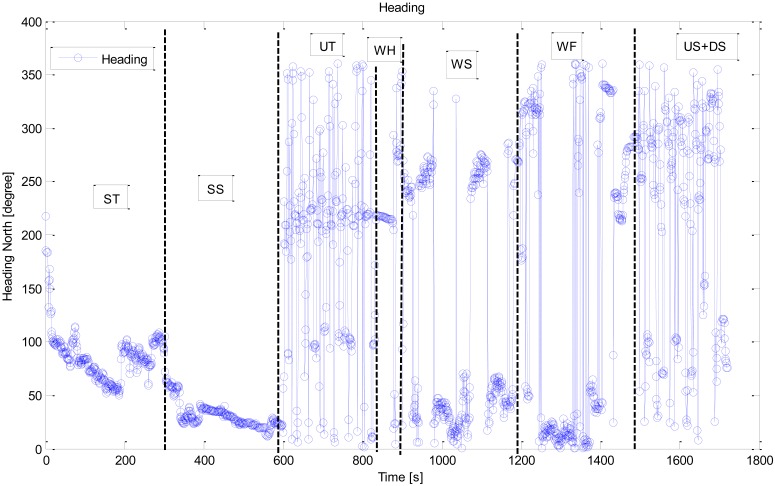
Magnetometer readings.

In addition to the accelerometer sensors, a magnetometer, also known as a digital compass, is another data source that can be utilized for motion recognition in a smartphone. The magnetometer, however, has some significant drawbacks. Indeed, magnetic disturbances are numerous, particularly in indoor environments. Figure 6 shows the corresponding heading readings from a built-in magnetometer for the same example readings presented above in Figure 5. Even though the user was in the ST state (0–200 s), the mean error introduced during this time period is still more than 20°. This large level of error means the magnetometer readings cannot be used directly for positioning in indoor navigation applications. Filtering and map matching are always demanded in the practical application. Instead of using the heading readings for navigation directly, in this paper we focus on applying them for recognizing the motion state of the user.

After analyzing the physical characteristics of the motion behavior, twenty-seven features are defined for the motion state estimation, including time-domain features of acceleration (Features 1–18) and heading (Features 19–21) and frequency domain features of acceleration (Features 22–27). Note that in Table 2 the dynamic acceleration denotes the real-time acceleration reading from smartphone minus the acceleration due to gravity.

## 4. LS-SVM Based Motion Recognition

The motion recognition method presented in this paper aims at determining which of the eight motions have caused the above twenty-seven simple features. The possible classification algorithms include k-Nearest Neighbour (kNN), Linear Discriminant Analysis (LDA), Quadratic Discriminant Analysis (QDA), Naïve Bayesian Classifier (NBC), Bayesian Network (BN), Decision Tree (DT), Artificial Neural Networks (ANN), Support Vector Machines (SVMs) and so forth. Thanks to the efficient pattern reorganization performance for the non-linear multi-class scenarios, in this study, we adopt the Least Square-Support Vector Machines (LS-SVM) [40] based classification algorithm, which is a class of kernel-based learning methods widely used for classification and regression analysis.

### 4.1. Support Vector Machines (SVMs)

**Figure 7.**
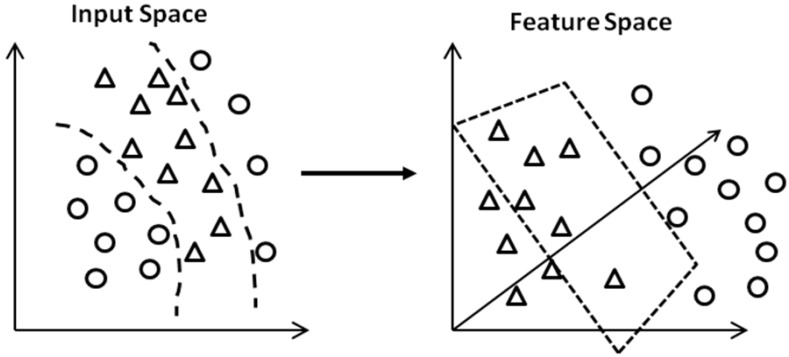
Mapping of the data from the input space to a high-dimensional feature space.

The concept of SVMs, which was originally developed for binary classification problems, is the use of hyperplanes to define decision boundaries separating data points of different classes. SVMs are able to handle both simple, linear classification tasks, as well as more complex, *i.e.*, nonlinear, classification problems [41]. The idea behind SVMs is to map the original data points from the input space to a high dimensional, or even infinite-dimensional, feature space such that the classification problem becomes simpler in the feature space, as shown in Figure 7.

When the data are linearly separable, the separating hyperplane can be defined in many ways. SVMs are based on the maximal margin principle, where the aim is to construct a hyperplane with maximal distance between the two classes. In most of real life applications, however, data of both classes overlap, which makes a perfect linear separation impossible. Therefore, a restricted number of misclassifications should be tolerated around the margins. The resulting optimization problem for SVMs, where violation of the constraints is penalized, is written as:

(5) $$\min_{\omega ,\xi ,b}J_{1}(\omega ,\xi )=\frac{1}{2}\omega^{T}\omega +C\sum_{i=1}^{N}\xi_{i}$$

such that:

(6) $$y_{i}(\omega^{T}\phi (x_{i})+b)\geq 1-\xi_{i},i=1,\ldots ,N,\xi_{i}\geq 0,i=1,\ldots ,N,$$

(7) $$y_{i}=\text{sign}(\omega^{T}\varphi (x_{i})+b$$

where *J_1_* is the cost function, *ω* is termed the weight vector, and the *C* is a positive regularization constant. The regularization constant in the cost function defines the tradeoff between a large margin and a misclassification error (*i.e.*, empirical risk minimization). SVMs are based on the principle of structural risk minimization which balances model complexity and empirical error. The first set of constraints corresponds to Equation (5), while the second set imposes positive slack variables *ξ_i_*, tolerating misclassifications. The value *ξ_i_* indicates the distance of *x_i_* with respect to the decision boundary, as follows:

*ξ_i_* ≥ 0: *y_i_*(*ω^T^φ*(*x_i_*) + *b*) < 0 implies that the decision function and the target have a different sign, indicating that *x_i_* is misclassified,
0 < *ξ_i_* < 1: *x_i_* is correctly classified, but lies inside the margin,
*ξ_i_* = 0: *x_i_* is correctly classified and lies outside the margin or on the margin boundary.

Typically, the problem formulation in Equations (5)–(7) is referred to as the primal optimization problem. Alternatively, however, the optimization problem for SVMs can be written in the dual space using the Lagrangian with Lagrange multipliers *α_i_* ≥ 0 for the first set of constraints shown in Equation (6). This alternative but equivalent formulation is useful in the cases where the original data are nonlinear.

The solution for the Lagrange multipliers is obtained by solving a quadratic programming problem. The SVM classifier takes the form:

(8) $$y(x)=\text{sign}(\sum_{i=1}^{N}\alpha_{i}y_{i}K(x,x_{i})+b)$$

where the kernel function *K*(·,·) is positive definite. It satisfies Mercer's condition then, meaning that *K*(*x,x_i_*) equals *φ*(*x*)*^T^φ*(*x_i_*). Using this so-called kernel trick, no explicit construction of the mapping *φ*(·) is needed. This enables SVMs to work in a high-dimensional (or infinite-dimensional) feature space, without actually performing calculations in this space. Various types of kernel functions can be chosen:

- (9) $$\text{Linear SVM}:K(x,z)=x^{T}z,$$
- (10) $$\text{Polynomial SVM of degree}\;d:K(x,z)={\left(\tau +x^{T}z\right)}^{d},\tau \geq 0$$
- (11) $$\text{Radial basis function}(\text{RBF}):K(x,z)=\text{exp}(-{\left\|x-z\right\|}_{2}^{2}/\delta^{2}),$$
- (12) $$\text{Multi-layer perceptron}(\text{MLP}):K(x,z)=\text{tanh}{\left(\kappa_{1}x^{T}z+\kappa_{2}\right)}^{d},$$

where *K*(·,·) is positive definite for all *δ* values in the RBF kernel case and *τ* ≥ 0 values in the polynomial case, but not for all possible choices of *κ_1_, κ_2_* in the MLP case. Such kernels transform the original data into a higher dimensional feature space, where they are separable by a hyperplane in this feature space.

### 4.2. LS-SVM

The classification technique used in this work is the LS-SVM. LS-SVM tackles linear systems rather than solving convex optimization problems, typically quadratic programs, as in standard support vector machines (SVM) [40]. This is done by both introducing a least squares loss function and working with equalities, instead of the intrinsic inequalities of SVM formulations. One advantage of this reformulation is complexity reduction. In the training phase, the LS-SVM classifier constructs a hyperplane in a high-dimensional space aiming to separate the data according to the different classes. This data separation should occur in such a way that the hyperplane has the largest distance to the nearest training data points of any class. These particular training data points define the so-called margin.

These parameters can be found by solving the following optimization problem having a quadratic cost function and equality constraints:

(13) $${\text{min}}_{\omega ,e,b}J_{2}(\omega ,e)=\frac{1}{2}\omega^{T}\omega +\frac{1}{2}\gamma \sum_{i=1}^{N}e_{i}$$

subject to:

(14) $$y_{i}(\omega^{T}\phi (x_{i})+b)\geq 1-e_{i},i=1,\ldots ,N$$

with *e* = [*e_1_*…*e_N_*]*^T^* a vector of error variables to tolerate misclassifications, *φ*(·): ℝ*^d^*→ℝ*^dh^* mapping from the input space into a high-dimensional (e.g., infinite dimensional) feature space of dimension *d_h_, ω* a vector of the same dimension as *φ*(·), *γ* is a positive regularization parameter, determining the trade-off between the margin size maximization and the training error minimization. *b* is a bias term. In this equation, the standard SVM formulation is modified using a least squares loss function with error variables *e_i_* and replacing the inequality constraints with equality constraints. Since, the value 1 in the equality constraints is a target value instead of a threshold value, the method is related to kernel Fisher discriminant analysis [40]. The Lagrangian for the problem in Equations (13) and (14) is:

(15) $$L(\omega ,b,e,\alpha )=J_{2}(\omega ,e)-\sum_{i=1}^{N}\alpha_{i}\left(y_{i}\left[\omega^{T}\phi (x_{i})+b\right]-1+e_{i}\right)$$

where *α* ∈ ℝ are the Lagrange multipliers, or support values.

Taking the conditions for optimality, we set:

(16) $$\left\{ \begin{array}{l} \frac{\partial L}{\partial \omega }=0\to \omega =\sum_{i=1}^{N}\alpha_{i}y_{i}\phi (x_{i}),\\ \frac{\partial L}{\partial b}=0\to \sum_{i=1}^{N}\alpha_{i}y_{i}=0,\\ \frac{\partial L}{\partial e_{i}}=0\to \alpha_{i}=\gamma e_{i},\\ \frac{\partial L}{\partial \alpha_{i}}=0\to y_{i}\left[\omega^{T}\phi (x_{i})+b\right]-1+e_{i}=0,i=1,\ldots ,N. \end{array}\right.$$

Whereas the primal problem is expressed in terms of the feature map, the linear optimization problem in the dual space is expressed in terms of the kernel function:

(17) $$\left(\begin{array}{ll} 0 & y^{T}\\ y & \Omega +\frac{1}{\gamma }I_{n} \end{array}\right)\left(\begin{array}{l} b\\ \alpha \end{array}\right)=\left(\begin{array}{l} 0\\ 1_{n} \end{array}\right)$$

where *y* = [*y_1_*…*y_N_*]*^T^α* = [*α_1_*…*α_N_*]*^T^* 1_n_ = [1…1]*^T^_1×N_* and Ω ∈ ℝ*^N×N^* is a matrix with elements Ω*_ij_* = *y_i_y_j_φ*(*x_i_*)*^T^φ*(*x_j_*), with *i, j* = 1, …, *N*. Provided with an input vector *x*, the resulting LS-SVM classifier in the dual space is similar to the standard SVM classifier:

(18) $$y(x)=\text{sign}\left(\sum_{i=1}^{N}\alpha_{i}y_{i}K(x,x_{i})+b\right)$$

where *K*(*x,x_i_*) = *φ*(*x*)*^T^φ*(*x_i_*) is a positive definite kernel matrix. The support values *α_i_* are proportional to the error of the corresponding training data points. This implies that usually every training data point is a support vector and no sparseness property remains in the LS-SVM formulation. However, high support values indicate a high contribution of the data point to the decision boundary. The choice of the regularization parameter and the kernel hyperparameter *δ* in case of an RBF kernel, is out of the scope for discussion in this paper. Hospodar gives an example of the kernel parameters selection in [42].

**Figure 8.**
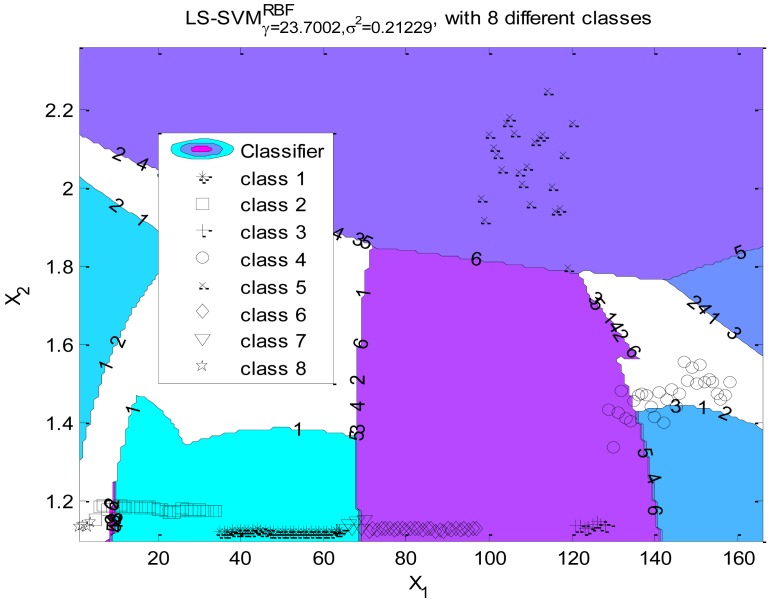
The projections of LS-SVM hyperlanes in the original feature space. (Class 1: ST, 2: SS, 3: WH, 4: WS, 5: WF, 6: UT, 7: US, 8: DS).

As shown in Figure 8, in this research we label the eight motion states as classes 1 to 8. These states are difficult to separate linearly in the original feature space. In order to classify the states, we apply a LS-SVM classifier using a RBF kernel function. The Nelder-Mead Simplex algorithm [43] is applied for tuning parameter optimization. The cross validation as a cost function is used for estimating the performance of the selected parameters [44]. As the optimized result, the regularization parameter γ equals 23.7002 and the kernel hyperparameter *δ^2^* equals 0.21229. Figure 8 presents the projection of the hyperplanes in the original feature space, which shows that the states are separable in the high dimensional feature space. The accuracy of the LS-SVM classifier for motion recognition will be fully discussed in Section 6.

## 5. Positioning Algorithms

In this paper, the motion recognition assisted indoor navigation solution interpolates the locations calculated by wireless positioning which uses the fingerprinting approach described below. Provided with the discrete locations from wireless positioning and recognized motion states, a grid-based filter based on the hidden Markov model is applied to compute the continuous positions of a smartphone user.

### 5.1. Fingerprinting Based Wireless Positioning

Received signal strength indicators (RSSIs) are the basic observables in this approach. The process consists of a training phase and a positioning phase. During the training phase, a radio map of probability distributions of the received signal strength is constructed for the targeted area. The targeted area is divided into a grid, and the central point of each cell in the grid is referred to as a reference point. The probability distribution of the received signal strength at each reference point is represented by a Weibull function [7], and the parameters of the Weibull function are estimated with the limited number of training samples.

During the positioning phase, the current location is determined using the measured RSSI observations in real time and the constructed radio map. The Bayesian theorem and Histogram Maximum Likelihood algorithm are used for positioning [45,46].

Given the RSSI measurement vector *O⃗*= {*O_1_, O_2_*… *O_k_*} from APs, the problem is to find the location *l* with the conditional probability *P*(*l*| *O⃗*) being maximized. Using the Bayesian theorem:

(19) $$\arg\max_{i}[P(l|\vec{O})]=\arg\max_{l}[\frac{P(\vec{O}|l)P(l)}{P(\vec{O})}]$$

where *P*(*O⃗*) is constant for all *l*. Therefore, the Equation (19) can be reduced as:

(20) $$\arg\max_{i}[P(l|\vec{O})]=\arg\max_{l}[P(\vec{O}|l)P(l)]$$

We assume that the mobile device has equal probability to access each reference point, thus *P*(*l*) can be considered as constant in this case, Equation (20) can be simplified as:

(21) $$\arg\max_{i}[P(l|\vec{O})]=\arg\max_{l}[P(\vec{O}|l)]$$

Now it becomes a problem of finding the maximum conditional probability of:

(22) $$P(\vec{O}|l)=\prod_{n=k}^{k}P(O_{n}|l)$$

where the conditional probability *P*(*O_n_* |*l*) is derived from the RSSI distribution stored in the fingerprint database.

## 5.2. Grid-Based Filter of HMM

The grid-based filter of hidden Markov model (HMM) is implemented to produce an optimal estimation based on the previous state. The transit probability matrix of HMM is computed according to the travelled distance which can be estimated by the knowledge about the motion over time. For instance, the travelled distance is zero if the current motion mode is static. The user travel distance while navigating can be calculated each second as:

(23) $$d_{t}=v_{t}\cdot t$$

where *v_t_* is the velocity output based on the recognized motion state and *t* is the time.

The velocity estimation models vary in different motion states. These estimations are out of the scope of this paper. More details about velocity estimation can be found in [27].

The grid-based filter produces an optimal estimation if the state space is discrete and consists of a finite number of states. If a numerical approximation is employed to obtain a discrete and finite state space, the grid-based filter produces a suboptimal estimation [47].

Given measurements up to epoch *t*-1, let the conditional probability of each state *S_i_* be denoted by *ω^i^_t-_*_1_*_|t-_*_1_, that is *ω^i^_t-_*_1_*_|t-_*_1_ *= P*(*X_t_*_-1_ = *S_i_*|*o*_1_,…,*o_t_*_-1_), *i* = 1,…, *N*. Then, given a hidden Markov model Λ = (*A,B,π*) [48], the posterior probability related to each state *S_i_* at epoch *t*-1 can be written as:

(24) $$P(X_{t-1)}=S_{j}|o_{1},\cdots ,o_{t-1})=\sum_{t=1}^{N}\omega_{t-1|t-1}^{i}\delta (S_{j}-S_{i}),j=1,\ldots ,N$$

where *δ*(·) is the Dirac delta function.

The grid-based filter consists of prediction and update stages as follows, similar to those used in other recursive Bayesian filters.

Prediction stage:

(25) $$P(X_{t}=S_{j}|o_{1},\cdots ,o_{t-1},\Lambda )=\sum_{t=1}^{N}\omega_{t|t-1}^{i}\delta (S_{j}-S_{i}),j=1,\ldots ,N$$

Update stage:

(26) $$P(X_{t}=S_{j}|o_{1},\cdots ,o_{t},\Lambda )=\sum_{t=1}^{N}\omega_{t|t}^{i}\delta (S_{j}-S_{i}),j=1,\ldots ,N$$

where

(27) $$\omega_{t|t-1}^{i}\equiv \sum_{j=1}^{N}\omega_{t-1|t-1}^{j}P(S_{t}^{i}|S_{t-1}^{j})$$

(28) $$\omega_{t|t}^{i}\equiv \frac{\omega_{t|t-1}^{i}P(o_{t}|S_{t}^{i})}{\sum_{j=1}^{N}\omega_{t|t-1}^{i}P(o_{t}|S_{t}^{j})}$$

Once the posterior probabilities of all states are estimated, the filter solution is given by the state with the maximum probability.

In the tests described in the following section, we applied magnetometer readings via map-matching instead of using the heading directly obtained from a magnetometer since the magnetic disturbances are numerous, particularly in indoor environments. With the heading input from the magnetometer and current position estimate, matched direction is derived from the segment vector in the topological network of the fingerprint database. In addition, the cumulative travel distance over the duration without WLAN positioning is used as an observation in the HMM grid-based filter for determining the position.

## 6. Test Results

To verify the solution proposed in this paper, some field tests were carried out in the Finnish Geodetic Institute (FGI) office building, which has three floors. A smartphone application was developed for collecting sensor data, labeling the motion state, and locating the smartphone position. Five persons collected the data for motion recognition in one day. Each person performed eight motion states respectively. Testers made marks at the beginning and end of the motion to separate the samples of motion states. All the collected data were divided into two groups. One was selected as a training data set. The other was utilized as a testing data set. The training data sets were used for learning the parameters of the classification algorithm. The testing data sets were used for validating the recognition rate of a classifier.

In order to evaluate the performance of LS-SVM classifier for motion recognition, the same data sets are also applied in four other classification algorithms for comparison: Bayesian Network using the Gaussian Mixture Model (BN-GMM), Decision Tree (DT), Linear Discriminant Analysis (LDA) and Quadratic Discriminant Analysis (QDA). Table 3 illustrates the recognition rates for the five classifiers with varying feature combinations. The bold and italic number indicates the best recognition rate in each feature combination in five classifiers. The bold and underlined number stands for the best recognition rate in each classifier.

The test results indicate that:

- LS-SVM classifier has the best performance in different feature combinations.
- Including all features does not help the recognition rate.
- Feature 4 and 5 are the most efficient features for the tested motion recognition.

**Figure 9.**
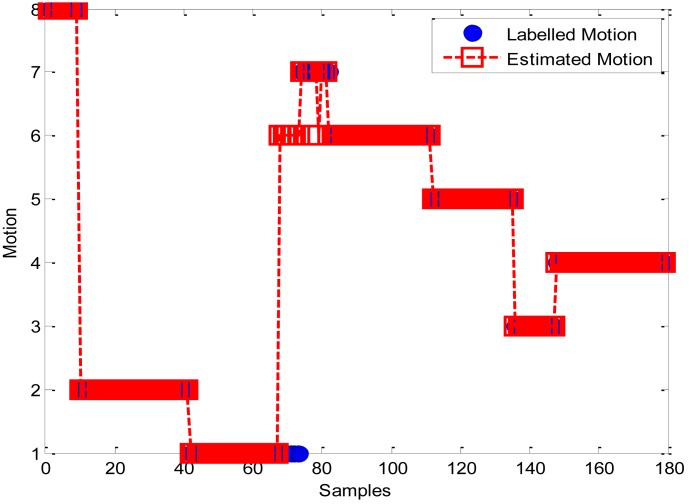
LS-SVM Motion state predictions (Motion state 1: ST, 2: SS, 3: WH, 4: WS, 5: WF, 6: UT, 7: US, 8: DS).

Another test was carried out by a tester who travelled around in the FGI office building for 20 min. As shown in Figure 9, eight motion states, including DS, SS, ST, US, UT, WF, WH, and WS, were performed in order. The confusion matrix for the motion recognition from the LS-SVM classifier is listed in Table 4. In the results for this test, 18.75% of ST motions and 22.22% of US motions are mistaken for UT using LS-SVM. The W-series motion states achieve a perfect success rate in this test.

To prove the advantage of wireless positioning combined with motion recognition, two positioning tests were conducted in the FGI building. A WLAN fingerprint database covers three floors of FGI was beforehand generated and used in the following tests. The first test, called a “Static Test,” was carried out in a static state—a user stood on a reference point while holding the phone in hand (ST) for ten minutes. The results are summarized in Table 5, where the average error is 3.43 m when the Maximum Likelihood (ML) algorithm was applied in wireless positioning. The mean error is reduced to 1.22 m when applying a motion-awareness assisted HMM algorithm. Furthermore, compared to the ML algorithm, the RMSE (Root Mean Square Error) and maximum error are all significantly decreased when using motion recognition assisted HMM algorithm.

The second test is called the “Stop-Go Test”. In the FGI office building, a tester stopped at each reference point to obtain the wireless positioning estimation, then moved to another reference point while randomly performing varying motion states between two stops. Table 6 compares the positioning results derived from ML and motion recognition assisted HMM algorithm. Some improvements can be found in motion recognition assisted HMM algorithm compared to the ML algorithm. Meanwhile, the motion recognition also raises the floor detection rate from 89.93% to 95.95%. The details are shown in Tables 7 and 8.

## 7. Conclusions and Future Work

In this paper, the motion recognition assisted wireless positioning method is presented. The raw data from the accelerometer and magnetometer on a smartphone are processed into twenty-seven features. Then eight motion states are predicted by separately applying the LS-SVM, BN-GMM, DT, LDA and QDA classifiers. The test results indicate that the LS-SVM classifier has an efficient performance of motion recognition rate compared with the other four classifiers. The recognition rates of T-series and V-series motions are lower than those of S-series and W-series motions. Furthermore, both positioning accuracy and floor detection rate are significantly improved by applying motion recognition in the wireless positioning algorithms.

Despite the fact that the motion recognition solution proposed in this paper provides correct motion recognition for up to 95.53% of the test cases, the motion behavior varies from person to person. In the future we will involve more persons for testing the motion recognition algorithms and determine the most useful features for classification. In addition, more motion states will be considered for indoor navigation. For instance, we currently only consider the “using-stairs” motion in the V-series motions. Other V-series motions such as “using-elevator” will be studied in the future. Lastly, the T-series motions introduce much more confusion because it is possible to combine them with the other motions simultaneously. Therefore, more efforts will be concentrated on the T-series motions in the future. For instance, we are currently studying the use of gyroscopes inside smartphones, which provide heading change rate.

## Figures and Tables

**Table 1.** Motion state definition.

**State**	**Series**	**Definition**
**ST**	S	A state where a user keeps a phone in hand without any movement.
**SS**	S	User's location does not change but the phone is in a swinging.
**WS**	W	Walking with a small arm swinging.
**WH**	W	Using the navigation application on the handset while walking.
**WF**	W	Fast walking with a significantly arm swinging.
**UT**	T	Making a U-turn.
**US**	V	Going up stairs.
**DS**	V	Going down stairs.

**Table 2.** Feature definition.

**Feature No.**	**Feature Name**	**Feature Definition**
**1**	MeanAccX	Mean value of the acceleration along x-axis.
**2**	MeanAccY	Mean value of the acceleration along y-axis.
**3**	MeanAccZ	Mean value of the acceleration along z-axis.
**4**	MeanAcc	Mean value of the acceleration.
**5**	MeanDynAccV	Mean value of the dynamic acceleration in the vertical plane.
**6**	MeanDynAccH	Mean value of the dynamic acceleration in the horizontal plane.
**7**	MeanAccH	Mean value of the horizontal acceleration.
**8**	MeanAccV	Mean value of the vertical acceleration minus gravity acceleration.
**9**	MeanDynAcc	Mean value of the dynamic acceleration.
**10**	VarAccX	Variance of the acceleration along x-axis.
**11**	VarAccY	Variance of the acceleration along y-axis.
**12**	VarAccZ	Variance of the acceleration along z-axis.
**13**	VarAcc	Variance of the acceleration.
**14**	VarDynAccV	Variance of the dynamic acceleration in the vertical plane.
**15**	VarDynAccH	Variance of the dynamic acceleration in the horizontal plane.
**16**	VarAccH	Variance of the horizontal acceleration.
**17**	VarAccV	Variance of the vertical acceleration.
**18**	VarDynAcc	Variance of the dynamic acceleration.
**19**	MeanMag	Mean value of the heading.
**20**	DiffMag	Heading change.
**21**	VarMag	Variance of the heading.
**22**	1stFreqAcc	1st dominant frequency of the acceleration.
**23**	Amp1stFreqAcc	Amplitude of the1st dominant frequency of the acceleration.
**24**	2ndFreqAcc	2nd dominant frequency of the acceleration.
**25**	Amp2ndFreqAcc	Amplitude of the 2nd dominant frequency of the acceleration.
**26**	FreqDiffAcc	Difference between two dominant frequencies.
**27**	AmpScaleAcc	Amplitude scale of two dominant frequencies.

**Table 3.** Classifier *vs.* Recognition Rate *vs.* Features.

**Classifier**	**Recognition Rate**				

	**LS-SVM**	**BN-GMM**	**DT**	**LDA**	**QDA**
**Feature 4**	87.15*^1^	67.04	77.66	75.98	74.30
**Feature 5**	***86.03***	53.07	56.43	62.01	63.13
**Feature 4,5**	***95.53***	73.74	83.80	86.59	86.03
**Feature 4,5,22**	***92.17***	79.33	88.83*^2^	**87.15**	86.03
**Feature 4,5,22,23**	***92.18***	**86.59**	88.83	84.36	83.24
**Feature 4,5,24,25**	***91.62***	64.80	85.48	73.74	null*^3^
**Feature 4,5,22,24**	***92.18***	75.98	88.83	74.86	null
**Feature 4,5,26**	***92.74***	73.18	85.48	73.18	87.71
**Feature 4,5,27**	***92.74***	78.21	83.80	83.24	**88.25**
**Feature 4,5,26,27**	***94.97***	77.10	85.48	77.10	null
**Feature 4,5,19,20,21**	***88.27***	68.72	86.03	83.24	null
**Feature 4,5,19,21**	***87.71***	74.86	82.12	84.92	null
**Feature 4,5,21**	***88.23***	77.10	83.24	87.15*^4^	80.45
**Feature 4,5,21,27**	***93.86***	81.01	84.92	86.03	null
**Feature 1,2,3**	***90.50***	67.60	65.36	null	null
**Feature 1,2,3,4,5**	***92.74***	76.54	82.68	null	null
**Feature 5,6,7,8,9**	***88.27***	72.07	82.12	null	null
**Feature 13,14**	***83.80***	47.49	76.54	64.25	64.80
**Feature 17,18**	***93.30***	48.04	70.39	59.78	68.72
**Feature 10-18**	80.45	51.40	***82.12***	null	null
**Feature 18**	***85.48***	42.46	53.07	52.51	58.10
**All Features**	53.63	***68.16***	64.25	null	null

*1 The bold and italic number indicates the best recognition rate in each feature combination.

*2 The bold and underlined number indicates the best recognition rate in each classifier.

*3 The null value is caused by the features which do not satisfy the requirements of the classifier.

*4 The recognition rates of combination feature 4, 5, 21 and feature 4, 5, 22 are the equally best in LDA classifier.

**Table 4.** Confusion Matrix for the motion recognition from LS-SVM classifier (Unit: %).

**Labelled Motion State**	**Recognized Motion State**							

	**ST**	**SS**	**WH**	**WS**	**WF**	**UT**	**US**	**DS**
**ST**	81.25	0	0	0	0	18.75	0	0
**SS**	0	100	0	0	0	0	0	0
**WH**	0	0	100	0	0	0	0	0
**WS**	0	0	0	100	0	0	0	0
**WF**	0	0	0	0	100	0	0	0
**UT**	0	0	0	0	0	100	0	0
**US**	0	0	0	0	0	22.22	77.78	0
**DS**	0	0	0	0	0	0	0	100

**Table 5.** Static Test (Unit: m).

**Static Test**	**ML**	**HMM (Motion-assisted)**
**Mean**	3.43	1.22
**RMSE**	5.98	2.55
**MaxErr**	21	9
**MinErr**	0	0

**Table 6.** Stop-Go Test (Unit: m).

**Stop-Go Test**	**ML**	**HMM (Motion- assisted)**
**Mean**	4.38	3.53
**RMSE**	6.02	4.55
**MaxErr**	18	9
**MinErr**	0	0

**Table 7.** Confusion matrix for floor detection using ML wireless positioning (Unit: %).

**Estimated Floor**	**Labelled Floor**		

	**1^st^**	**2^nd^**	**3^rd^**
**1^st^**	93.94	6.06	0
**2^nd^**	4.00	92.00	4.00
**3^rd^**	0	17.95	82.05

**Table 8.** Confusion matrix for floor detection using motion-assisted HMM wireless positioning (Unit: %).

**Estimated Floor**	**Labelled Floor**		

	**1^st^**	**2^nd^**	**3^rd^**
**1^st^**	96.97	3.03	0
**2^nd^**	4.00	96.00	0
**3^rd^**	0	5.13	94.87
